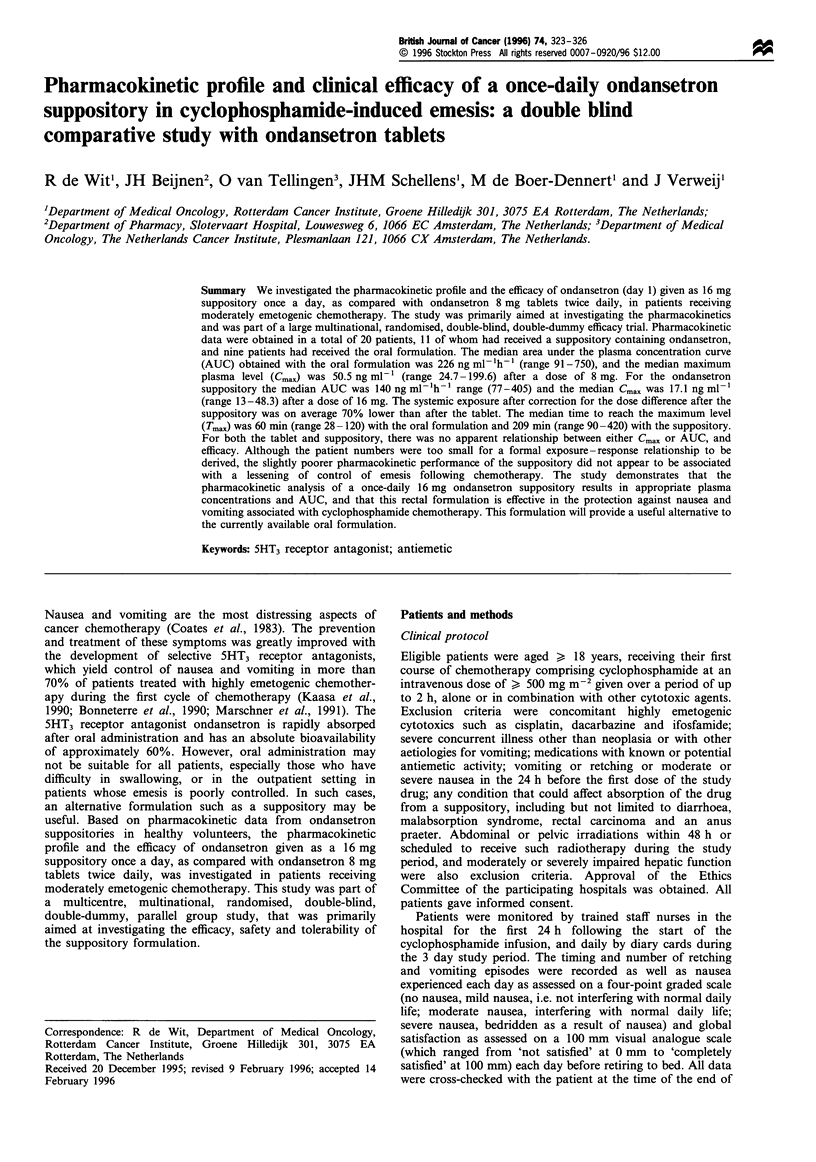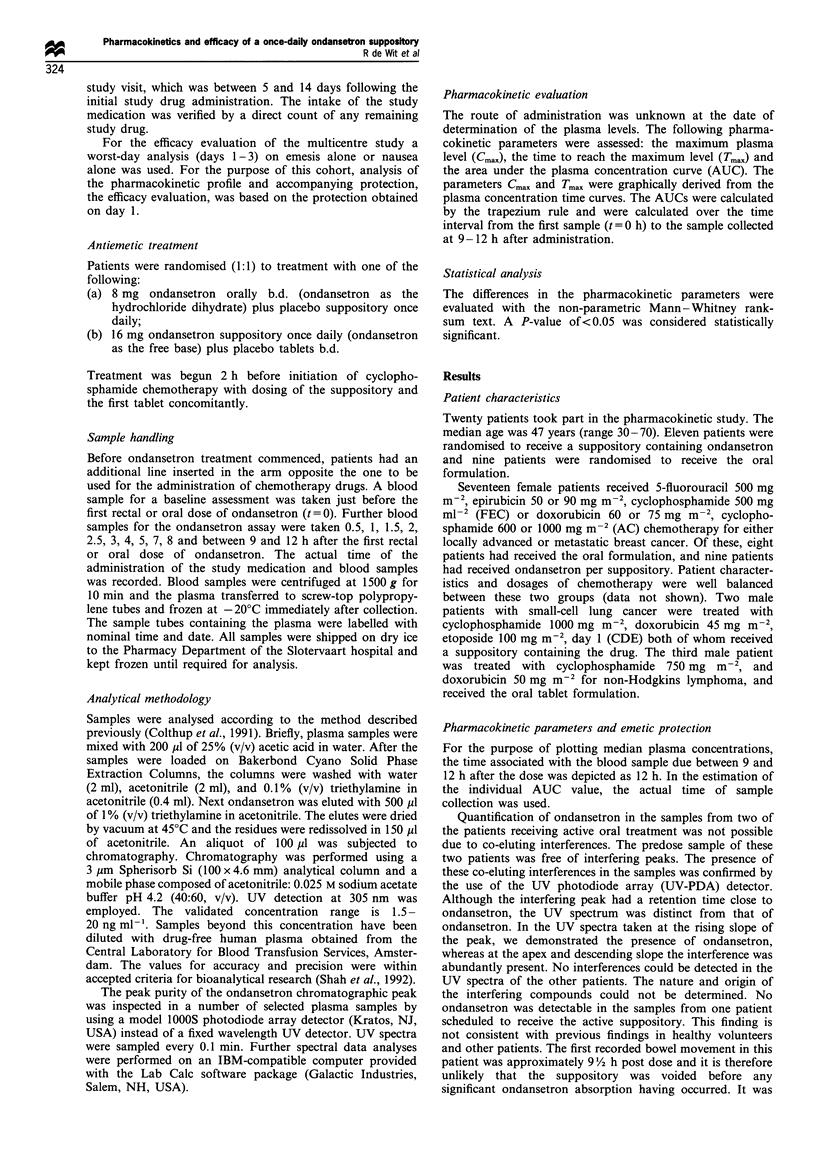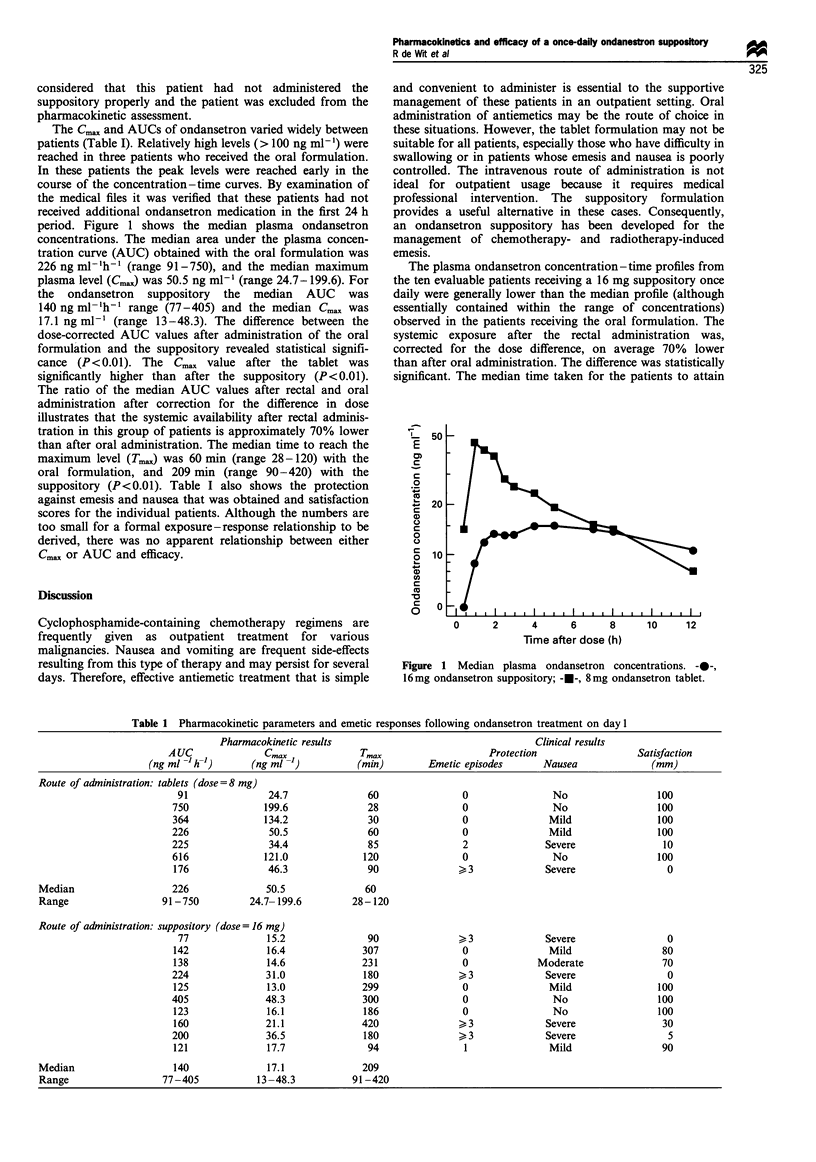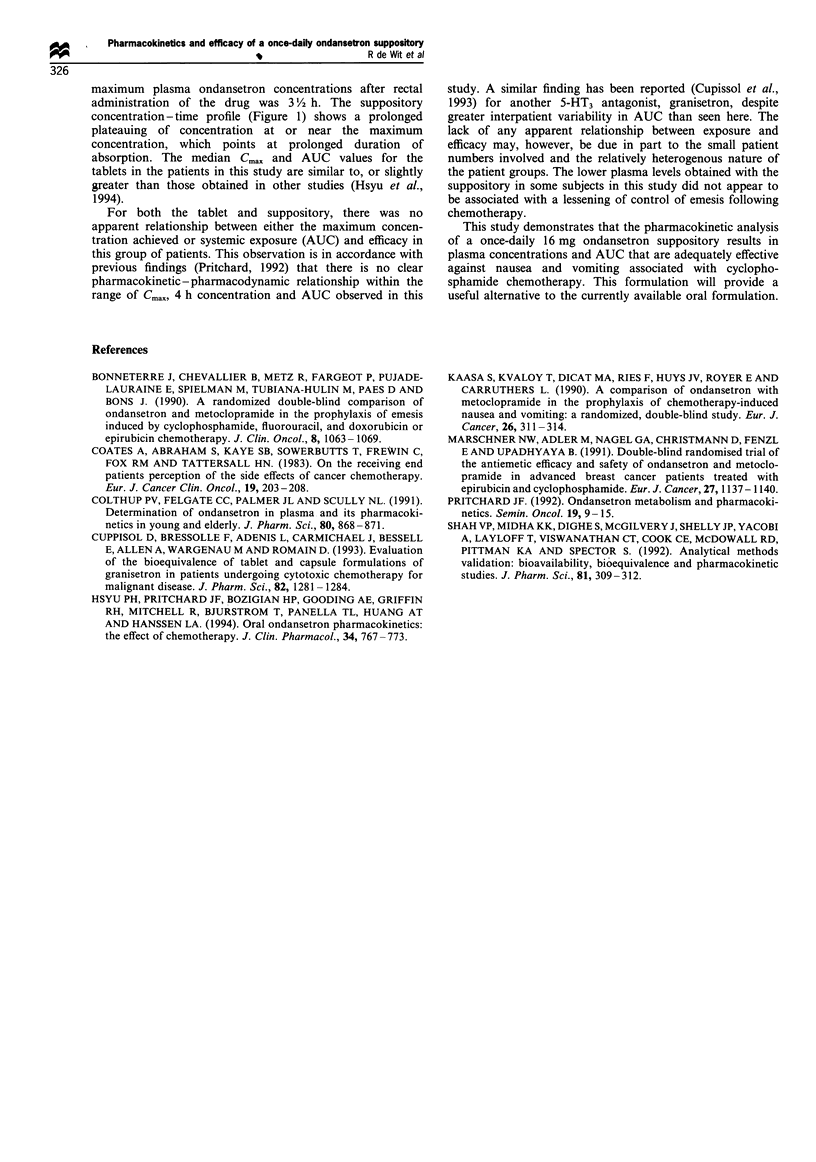# Pharmacokinetic profile and clinical efficacy of a once-daily ondansetron suppository in cyclophosphamide-induced emesis: a double blind comparative study with ondansetron tablets.

**DOI:** 10.1038/bjc.1996.361

**Published:** 1996-07

**Authors:** R. de Wit, J. H. Beijnen, O. van Tellingen, J. H. Schellens, M. de Boer-Dennert, J. Verweij

**Affiliations:** Department of Medical Oncology, Rotterdam Cancer Institute, The Netherlands.

## Abstract

We investigated the pharmacokinetic profile and the efficacy of ondansetron (day 1) given as 16 mg suppository once a day, as compared with ondansetron 8 mg tablets twice daily, in patients receiving moderately emetogenic chemotherapy. The study was primarily aimed at investigating the pharmacokinetics and was part of a large multinational, randomised, double-blind, double-dummy efficacy trial. Pharmacokinetic data were obtained in a total of 20 patients, 11 of whom had received a suppository containing ondansetron, and nine patients had received the oral formulation. The median area under the plasma concentration curve (AUC) obtained with the oral formulation was 226 ng ml-1h-1 (range 91-750), and the median maximum plasma level (Cmax) was 50.5 ng ml-1 (range 24.7-199.6) after a dose of 8 mg. For the ondansetron suppository the median AUC was 140 ng ml-1h-1 range (77-405) and the median Cmax was 17.1 ng ml-1 (range 13-48.3) after a dose of 16 mg. The systemic exposure after correction for the dose difference after the suppository was on average 70% lower than after the tablet. The median time to reach the maximum level (Tmax) was 60 min (range 28-120) with the oral formulation and 209 min (range 90-420) with the suppository. For both the tablet and suppository, there was no apparent relationship between either Cmax or AUC, and efficacy. Although the patient numbers were too small for a formal exposure-response relationship to be derived, the slightly poorer pharmacokinetic performance of the suppository did not appear to be associated with a lessening of control of emesis following chemotherapy. The study demonstrates that the pharmacokinetic analysis of a once-daily 16 mg ondansetron suppository results in appropriate plasma concentrations and AUC, and that this rectal formulation is effective in the protection against nausea and vomiting associated with cyclophosphamide chemotherapy. This formulation will provide a useful alternative to the currently available oral formulation.


British Journal of Cancer (1996) 74, 323-326
© 1996 Stockton Press All rights reserved 0007-0920/96 $12.00

### Pharmacokinetic profile and clinical efficacy of a once-daily ondansetron suppository in cyclophosphamide-induced emesis: a double blind comparative study with ondansetron tablets

R de Wit[1], JH Beijnen[2], O van Tellingen[3], JHM Schellens[1], M de Boer-Dennert[1] and J Verweij[1]

[1]*Department of Medical Oncology, Rotterdam Cancer Institute, Groene Hilledijk 301, 3075 EA Rotterdam, The Netherlands;* [2]*Department of Pharmacy, Slotervaart Hospital, Louwesweg 6, 1066 EC Amsterdam, The Netherlands;* [3]*Department of Medical Oncology, The Netherlands Cancer Institute, Plesmanlaan 121, 1066 CX Amsterdam, The Netherlands.*

**Summary** We investigated the pharmacokinetic profile and the efficacy of ondansetron (day 1) given as 16 mg suppository once a day, as compared with ondansetron 8 mg tablets twice daily, in patients receiving moderately emetogenic chemotherapy. The study was primarily aimed at investigating the pharmacokinetics and was part of a large multinational, randomised, double-blind, double-dummy efficacy trial. Pharmacokinetic data were obtained in a total of 20 patients, 11 of whom had received a suppository containing ondansetron, and nine patients had received the oral formulation. The median area under the plasma concentration curve (AUC) obtained with the oral formulation was 226 ng $ml^{-1}h^{-1}$ (range 91–750), and the median maximum plasma level ($C_{max}$) was 50.5 ng $ml^{-1}$ (range 24.7–199.6) after a dose of 8 mg. For the ondansetron suppository the median AUC was 140 ng $ml^{-1}h^{-1}$ range (77–405) and the median $C_{max}$ was 17.1 ng $ml^{-1}$ (range 13–48.3) after a dose of 16 mg. The systemic exposure after correction for the dose difference after the suppository was on average 70% lower than after the tablet. The median time to reach the maximum level ($T_{max}$) was 60 min (range 28–120) with the oral formulation and 209 min (range 90–420) with the suppository. For both the tablet and suppository, there was no apparent relationship between either $C_{max}$ or AUC, and efficacy. Although the patient numbers were too small for a formal exposure–response relationship to be derived, the slightly poorer pharmacokinetic performance of the suppository did not appear to be associated with a lessening of control of emesis following chemotherapy. The study demonstrates that the pharmacokinetic analysis of a once-daily 16 mg ondansetron suppository results in appropriate plasma concentrations and AUC, and that this rectal formulation is effective in the protection against nausea and vomiting associated with cyclophosphamide chemotherapy. This formulation will provide a useful alternative to the currently available oral formulation.

**Keywords:** $5HT_3$ receptor antagonist; antiemetic

Nausea and vomiting are the most distressing aspects of cancer chemotherapy (Coates *et al.*, 1983). The prevention and treatment of these symptoms was greatly improved with the development of selective $5HT_3$ receptor antagonists, which yield control of nausea and vomiting in more than 70% of patients treated with highly emetogenic chemotherapy during the first cycle of chemotherapy (Kaasa *et al.*, 1990; Bonneterre *et al.*, 1990; Marschner *et al.*, 1991). The $5HT_3$ receptor antagonist ondansetron is rapidly absorped after oral administration and has an absolute bioavailability of approximately 60%. However, oral administration may not be suitable for all patients, especially those who have difficulty in swallowing, or in the outpatient setting in patients whose emesis is poorly controlled. In such cases, an alternative formulation such as a suppository may be useful. Based on pharmacokinetic data from ondansetron suppositories in healthy volunteers, the pharmacokinetic profile and the efficacy of ondansetron given as a 16 mg suppository once a day, as compared with ondansetron 8 mg tablets twice daily, was investigated in patients receiving moderately emetogenic chemotherapy. This study was part of a multicentre, multinational, randomised, double-blind, double-dummy, parallel group study, that was primarily aimed at investigating the efficacy, safety and tolerability of the suppository formulation.

Correspondence: R de Wit, Department of Medical Oncology, Rotterdam Cancer Institute, Groene Hilledijk 301, 3075 EA Rotterdam, The Netherlands
Received 20 December 1995; revised 9 February 1996; accepted 14 February 1996

### Patients and methods

### *Clinical protocol*

Eligible patients were aged ⩾ 18 years, receiving their first course of chemotherapy comprising cyclophosphamide at an intravenous dose of ⩾ 500 mg $m^{-2}$ given over a period of up to 2 h, alone or in combination with other cytotoxic agents. Exclusion criteria were concomitant highly emetogenic cytotoxics such as cisplatin, dacarbazine and ifosfamide; severe concurrent illness other than neoplasia or with other aetiologies for vomiting; medications with known or potential antiemetic activity; vomiting or retching or moderate or severe nausea in the 24 h before the first dose of the study drug; any condition that could affect absorption of the drug from a suppository, including but not limited to diarrhoea, malabsorption syndrome, rectal carcinoma and an anus praeter. Abdominal or pelvic irradiations within 48 h or scheduled to receive such radiotherapy during the study period, and moderately or severely impaired hepatic function were also exclusion criteria. Approval of the Ethics Committee of the participating hospitals was obtained. All patients gave informed consent.

Patients were monitored by trained staff nurses in the hospital for the first 24 h following the start of the cyclophosphamide infusion, and daily by diary cards during the 3 day study period. The timing and number of retching and vomiting episodes were recorded as well as nausea experienced each day as assessed on a four-point graded scale (no nausea, mild nausea, i.e. not interfering with normal daily life; moderate nausea, interfering with normal daily life; severe nausea, bedridden as a result of nausea) and global satisfaction as assessed on a 100 mm visual analogue scale (which ranged from 'not satisfied' at 0 mm to 'completely satisfied' at 100 mm) each day before retiring to bed. All data were cross-checked with the patient at the time of the end of study visit, which was between 5 and 14 days following the initial study drug administration. The intake of the study medication was verified by a direct count of any remaining study drug.

For the efficacy evaluation of the multicentre study a worst-day analysis (days 1–3) on emesis alone or nausea alone was used. For the purpose of this cohort, analysis of the pharmacokinetic profile and accompanying protection, the efficacy evaluation, was based on the protection obtained on day 1.

### *Antiemetic treatment*

Patients were randomised (1:1) to treatment with one of the following:

(a) 8 mg ondansetron orally b.d. (ondansetron as the hydrochloride dihydrate) plus placebo suppository once daily;
(b) 16 mg ondansetron suppository once daily (ondansetron as the free base) plus placebo tablets b.d.

Treatment was begun 2 h before initiation of cyclophosphamide chemotherapy with dosing of the suppository and the first tablet concomitantly.

### *Sample handling*

Before ondansetron treatment commenced, patients had an additional line inserted in the arm opposite the one to be used for the administration of chemotherapy drugs. A blood sample for a baseline assessment was taken just before the first rectal or oral dose of ondansetron ($t=0$). Further blood samples for the ondansetron assay were taken 0.5, 1, 1.5, 2, 2.5, 3, 4, 5, 7, 8 and between 9 and 12 h after the first rectal or oral dose of ondansetron. The actual time of the administration of the study medication and blood samples was recorded. Blood samples were centrifuged at 1500 *g* for 10 min and the plasma transferred to screw-top polypropylene tubes and frozen at −20°C immediately after collection. The sample tubes containing the plasma were labelled with nominal time and date. All samples were shipped on dry ice to the Pharmacy Department of the Slotervaart hospital and kept frozen until required for analysis.

### *Analytical methodology*

Samples were analysed according to the method described previously (Colthup *et al.*, 1991). Briefly, plasma samples were mixed with 200 $\mu$l of 25% (v/v) acetic acid in water. After the samples were loaded on Bakerbond Cyano Solid Phase Extraction Columns, the columns were washed with water (2 ml), acetonitrile (2 ml), and 0.1% (v/v) triethylamine in acetonitrile (0.4 ml). Next ondansetron was eluted with 500 $\mu$l of 1% (v/v) triethylamine in acetonitrile. The elutes were dried by vacuum at 45°C and the residues were redissolved in 150 $\mu$l of acetonitrile. An aliquot of 100 $\mu$l was subjected to chromatography. Chromatography was performed using a 3 $\mu$m Spherisorb Si (100 × 4.6 mm) analytical column and a mobile phase composed of acetonitrile: 0.025 M sodium acetate buffer pH 4.2 (40:60, v/v). UV detection at 305 nm was employed. The validated concentration range is 1.5–20 ng ml$^{-1}$. Samples beyond this concentration have been diluted with drug-free human plasma obtained from the Central Laboratory for Blood Transfusion Services, Amsterdam. The values for accuracy and precision were within accepted criteria for bioanalytical research (Shah *et al.*, 1992).

The peak purity of the ondansetron chromatographic peak was inspected in a number of selected plasma samples by using a model 1000S photodiode array detector (Kratos, NJ, USA) instead of a fixed wavelength UV detector. UV spectra were sampled every 0.1 min. Further spectral data analyses were performed on an IBM-compatible computer provided with the Lab Calc software package (Galactic Industries, Salem, NH, USA).

### *Pharmacokinetic evaluation*

The route of administration was unknown at the date of determination of the plasma levels. The following pharmacokinetic parameters were assessed: the maximum plasma level ($C_{max}$), the time to reach the maximum level ($T_{max}$) and the area under the plasma concentration curve (AUC). The parameters $C_{max}$ and $T_{max}$ were graphically derived from the plasma concentration time curves. The AUCs were calculated by the trapezium rule and were calculated over the time interval from the first sample ($t=0$ h) to the sample collected at 9–12 h after administration.

### *Statistical analysis*

The differences in the pharmacokinetic parameters were evaluated with the non-parametric Mann–Whitney rank-sum text. A *P*-value of $<0.05$ was considered statistically significant.

### Results

### *Patient characteristics*

Twenty patients took part in the pharmacokinetic study. The median age was 47 years (range 30–70). Eleven patients were randomised to receive a suppository containing ondansetron and nine patients were randomised to receive the oral formulation.

Seventeen female patients received 5-fluorouracil 500 mg m$^{-2}$, epirubicin 50 or 90 mg m$^{-2}$, cyclophosphamide 500 mg ml$^{-2}$ (FEC) or doxorubicin 60 or 75 mg m$^{-2}$, cyclophosphamide 600 or 1000 mg m$^{-2}$ (AC) chemotherapy for either locally advanced or metastatic breast cancer. Of these, eight patients had received the oral formulation, and nine patients had received ondansetron per suppository. Patient characteristics and dosages of chemotherapy were well balanced between these two groups (data not shown). Two male patients with small-cell lung cancer were treated with cyclophosphamide 1000 mg m$^{-2}$, doxorubicin 45 mg m$^{-2}$, etoposide 100 mg m$^{-2}$, day 1 (CDE) both of whom received a suppository containing the drug. The third male patient was treated with cyclophosphamide 750 mg m$^{-2}$, and doxorubicin 50 mg m$^{-2}$ for non-Hodgkins lymphoma, and received the oral tablet formulation.

### *Pharmacokinetic parameters and emetic protection*

For the purpose of plotting median plasma concentrations, the time associated with the blood sample due between 9 and 12 h after the dose was depicted as 12 h. In the estimation of the individual AUC value, the actual time of sample collection was used.

Quantification of ondansetron in the samples from two of the patients receiving active oral treatment was not possible due to co-eluting interferences. The predose sample of these two patients was free of interfering peaks. The presence of these co-eluting interferences in the samples was confirmed by the use of the UV photodiode array (UV-PDA) detector. Although the interfering peak had a retention time close to ondansetron, the UV spectrum was distinct from that of ondansetron. In the UV spectra taken at the rising slope of the peak, we demonstrated the presence of ondansetron, whereas at the apex and descending slope the interference was abundantly present. No interferences could be detected in the UV spectra of the other patients. The nature and origin of the interfering compounds could not be determined. No ondansetron was detectable in the samples from one patient scheduled to receive the active suppository. This finding is not consistent with previous findings in healthy volunteers and other patients. The first recorded bowel movement in this patient was approximately 9½ h post dose and it is therefore unlikely that the suppository was voided before any significant ondansetron absorption having occurred. It was considered that this patient had not administered the suppository properly and the patient was excluded from the pharmacokinetic assessment.

The $C_{max}$ and AUCs of ondansetron varied widely between patients (Table I). Relatively high levels (> 100 ng $ml^{-1}$) were reached in three patients who received the oral formulation. In these patients the peak levels were reached early in the course of the concentration–time curves. By examination of the medical files it was verified that these patients had not received additional ondansetron medication in the first 24 h period. Figure 1 shows the median plasma ondansetron concentrations. The median area under the plasma concentration curve (AUC) obtained with the oral formulation was 226 ng $ml^{-1}h^{-1}$ (range 91–750), and the median maximum plasma level ($C_{max}$) was 50.5 ng $ml^{-1}$ (range 24.7–199.6). For the ondansetron suppository the median AUC was 140 ng $ml^{-1}h^{-1}$ range (77–405) and the median $C_{max}$ was 17.1 ng $ml^{-1}$ (range 13–48.3). The difference between the dose-corrected AUC values after administration of the oral formulation and the suppository revealed statistical significance ($P<0.01$). The $C_{max}$ value after the tablet was significantly higher than after the suppository ($P<0.01$). The ratio of the median AUC values after rectal and oral administration after correction for the difference in dose illustrates that the systemic availability after rectal administration in this group of patients is approximately 70% lower than after oral administration. The median time to reach the maximum level ($T_{max}$) was 60 min (range 28–120) with the oral formulation, and 209 min (range 90–420) with the suppository ($P<0.01$). Table I also shows the protection against emesis and nausea that was obtained and satisfaction scores for the individual patients. Although the numbers are too small for a formal exposure–response relationship to be derived, there was no apparent relationship between either $C_{max}$ or AUC and efficacy.

### Discussion

Cyclophosphamide-containing chemotherapy regimens are frequently given as outpatient treatment for various malignancies. Nausea and vomiting are frequent side-effects resulting from this type of therapy and may persist for several days. Therefore, effective antiemetic treatment that is simple and convenient to administer is essential to the supportive management of these patients in an outpatient setting. Oral administration of antiemetics may be the route of choice in these situations. However, the tablet formulation may not be suitable for all patients, especially those who have difficulty in swallowing or in patients whose emesis and nausea is poorly controlled. The intravenous route of administration is not ideal for outpatient usage because it requires medical professional intervention. The suppository formulation provides a useful alternative in these cases. Consequently, an ondansetron suppository has been developed for the management of chemotherapy- and radiotherapy-induced emesis.

The plasma ondansetron concentration–time profiles from the ten evaluable patients receiving a 16 mg suppository once daily were generally lower than the median profile (although essentially contained within the range of concentrations) observed in the patients receiving the oral formulation. The systemic exposure after the rectal administration was, corrected for the dose difference, on average 70% lower than after oral administration. The difference was statistically significant. The median time taken for the patients to attain

**Figure 1** Median plasma ondansetron concentrations. -●-, 16 mg ondansetron suppository; -■-, 8 mg ondansetron tablet.

**Table 1** Pharmacokinetic parameters and emetic responses following ondansetron treatment on day 1

| | *Pharmacokinetic results* | | | *Clinical results* | | |
|---|---|---|---|---|---|---|
| | *AUC (ng ml$^{-1}$ h$^{-1}$)* | *$C_{max}$ (ng ml$^{-1}$)* | *$T_{max}$ (min)* | *Protection: Emetic episodes* | *Protection: Nausea* | *Satisfaction (mm)* |
| *Route of administration: tablets (dose = 8 mg)* | | | | | | |
| | 91 | 24.7 | 60 | 0 | No | 100 |
| | 750 | 199.6 | 28 | 0 | No | 100 |
| | 364 | 134.2 | 30 | 0 | Mild | 100 |
| | 226 | 50.5 | 60 | 0 | Mild | 100 |
| | 225 | 34.4 | 85 | 2 | Severe | 10 |
| | 616 | 121.0 | 120 | 0 | No | 100 |
| | 176 | 46.3 | 90 | ⩾3 | Severe | 0 |
| Median | 226 | 50.5 | 60 | | | |
| Range | 91–750 | 24.7–199.6 | 28–120 | | | |
| *Route of administration: suppository (dose = 16 mg)* | | | | | | |
| | 77 | 15.2 | 90 | ⩾3 | Severe | 0 |
| | 142 | 16.4 | 307 | 0 | Mild | 80 |
| | 138 | 14.6 | 231 | 0 | Moderate | 70 |
| | 224 | 31.0 | 180 | ⩾3 | Severe | 0 |
| | 125 | 13.0 | 299 | 0 | Mild | 100 |
| | 405 | 48.3 | 300 | 0 | No | 100 |
| | 123 | 16.1 | 186 | 0 | No | 100 |
| | 160 | 21.1 | 420 | ⩾3 | Severe | 30 |
| | 200 | 36.5 | 180 | ⩾3 | Severe | 5 |
| | 121 | 17.7 | 94 | 1 | Mild | 90 |
| Median | 140 | 17.1 | 209 | | | |
| Range | 77–405 | 13–48.3 | 91–420 | | | |

maximum plasma ondansetron concentrations after rectal administration of the drug was 3½ h. The suppository concentration–time profile (Figure 1) shows a prolonged plateauing of concentration at or near the maximum concentration, which points at prolonged duration of absorption. The median $C_{max}$ and AUC values for the tablets in the patients in this study are similar to, or slightly greater than those obtained in other studies (Hsyu *et al.*, 1994).

For both the tablet and suppository, there was no apparent relationship between either the maximum concentration achieved or systemic exposure (AUC) and efficacy in this group of patients. This observation is in accordance with previous findings (Pritchard, 1992) that there is no clear pharmacokinetic–pharmacodynamic relationship within the range of $C_{max}$, 4 h concentration and AUC observed in this study. A similar finding has been reported (Cupissol *et al.*, 1993) for another 5-$HT_3$ antagonist, granisetron, despite greater interpatient variability in AUC than seen here. The lack of any apparent relationship between exposure and efficacy may, however, be due in part to the small patient numbers involved and the relatively heterogenous nature of the patient groups. The lower plasma levels obtained with the suppository in some subjects in this study did not appear to be associated with a lessening of control of emesis following chemotherapy.

This study demonstrates that the pharmacokinetic analysis of a once-daily 16 mg ondansetron suppository results in plasma concentrations and AUC that are adequately effective against nausea and vomiting associated with cyclophosphamide chemotherapy. This formulation will provide a useful alternative to the currently available oral formulation.

### References

BONNETERRE J, CHEVALLIER B, METZ R, FARGEOT P, PUJADE-LAURAINE E, SPIELMAN M, TUBIANA-HULIN M, PAES D AND BONS J. (1990). A randomized double-blind comparison of ondansetron and metoclopramide in the prophylaxis of emesis induced by cyclophosphamide, fluorouracil, and doxorubicin or epirubicin chemotherapy. *J. Clin. Oncol.*, **8**, 1063–1069.

COATES A, ABRAHAM S, KAYE SB, SOWERBUTTS T, FREWIN C, FOX RM AND TATTERSALL HN. (1983). On the receiving end patients perception of the side effects of cancer chemotherapy. *Eur. J. Cancer Clin. Oncol.*, **19**, 203–208.

COLTHUP PV, FELGATE CC, PALMER JL AND SCULLY NL. (1991). Determination of ondansetron in plasma and its pharmacokinetics in young and elderly. *J. Pharm. Sci.*, **80**, 868–871.

CUPPISOL D, BRESSOLLE F, ADENIS L, CARMICHAEL J, BESSELL E, ALLEN A, WARGENAU M AND ROMAIN D. (1993). Evaluation of the bioequivalence of tablet and capsule formulations of granisetron in patients undergoing cytotoxic chemotherapy for malignant disease. *J. Pharm. Sci.*, **82**, 1281–1284.

HSYU PH, PRITCHARD JF, BOZIGIAN HP, GOODING AE, GRIFFIN RH, MITCHELL R, BJURSTROM T, PANELLA TL, HUANG AT AND HANSSEN LA. (1994). Oral ondansetron pharmacokinetics: the effect of chemotherapy. *J. Clin. Pharmacol.*, **34**, 767–773.

KAASA S, KVALOY T, DICAT MA, RIES F, HUYS JV, ROYER E AND CARRUTHERS L. (1990). A comparison of ondansetron with metoclopramide in the prophylaxis of chemotherapy-induced nausea and vomiting: a randomized, double-blind study. *Eur. J. Cancer*, **26**, 311–314.

MARSCHNER NW, ADLER M, NAGEL GA, CHRISTMANN D, FENZL E AND UPADHYAYA B. (1991). Double-blind randomised trial of the antiemetic efficacy and safety of ondansetron and metoclopramide in advanced breast cancer patients treated with epirubicin and cyclophosphamide. *Eur. J. Cancer*, **27**, 1137–1140.

PRITCHARD JF. (1992). Ondansetron metabolism and pharmacokinetics. *Semin. Oncol.* **19**, 9–15.

SHAH VP, MIDHA KK, DIGHE S, MCGILVERY J, SHELLY JP, YACOBI A, LAYLOFF T, VISWANATHAN CT, COOK CE, MCDOWALL RD, PITTMAN KA AND SPECTOR S. (1992). Analytical methods validation: bioavailability, bioequivalence and pharmacokinetic studies. *J. Pharm. Sci.*, **81**, 309–312.